# Replacing the 238th aspartic acid with an arginine impaired the oligomerization activity and inflammation-inducing property of pyolysin

**DOI:** 10.1080/21505594.2018.1491256

**Published:** 2018-08-01

**Authors:** Wenlong Zhang, Haili Wang, Bing Wang, Yue Zhang, Yunhao Hu, Bo Ma, Junwei Wang

**Affiliations:** a College of Veterinary Medicine, Northeast Agricultural University, Harbin, PR China; b China Ministry of Agriculture Key Laboratory of Animal Pathogen Biology, Northeastern Science Inspection Station, Harbin, PR China

**Keywords:** *Trueperella pyogenes*, pyolysin, oligomerization, pore-forming activity, inflammation response

## Abstract

*Trueperella pyogenes* (*T. pyogenes*) is an important opportunistic pathogen. Pyolysin (PLO) importantly contributes to the pathogenicity of *T. pyogenes*. However, the relationship between the structure and function and the virulence of PLO is not well documented. In the current study, recombinant PLO (rPLO) and three rPLO mutants were prepared. rPLO D238R, a mutant with the 238th aspartic acid replaced with an arginine, showed impairment in oligomerization activity on cholesterol-containing liposome and pore-forming activity on sheep red blood cell membrane. Further study employing the prepared mutants confirmed that the pore-forming activity of PLO is essential for inducing excessive inflammation responses in mice by upregulating the expression levels of IL-1β, TNF-α, and IL-6. By contrast, rPLO P499F, another mutant with impaired cell membrane binding capacity, elicited an inflammation response that was dependent on pathogen-associated molecular pattern (PAMP) activity, given that the mutant significantly upregulated the expression of IL-10 in macrophages and in mice, whereas rPLO did not. Results indicated that domain 1 of the PLO molecule plays an important role in maintaining pore-forming activity. Moreover, the PLO pore-forming activity and not PAMP activity is responsible for the inflammation-inducing effect of PLO. The results of this study provided new information for research field on the structure, function, and virulence of PLO.

**Abbreviations**: *T. pyogenes: Trueperella pyogenes*; PLO: Pyolysin; rPLO: recombinant PLO; PAMP: pathogen-associated molecular pattern; CDCs: cholesterol-dependent cytolysins; PLY: pneumolysin; NLRP3: NLR family pyrin domain containing protein 3; PRRs: pattern recognition receptors; Asp: aspartic acid; TLR4: Toll-like receptor 4; Arg: arginine; Asn: asparagine; IPTG: Isopropyl-β-d-thiogalactoside; PBS: phosphate-buffered saline; sRBCs: sheep red blood cells; TEM: Transmission electron microscopy; RBCM: red blood cell membrane; SDS-PAGE: sodium dodecyl sulfate–polyacrylamide gel electrophoresis; NC membrane: nitrocellulose membrane; SDS-AGE: dodecyl sulfate agarose gel electrophoresis; MDBK cells: Madin–Darby bovine kidney cells; RPMI-1640 medium: Roswell Park Memorial Institute-1640 medium; FBS: fetal bovine serum; BMDMs: bone marrow-derived macrophages; TNF-α: tumor necrosis factor α; IL-1β: interleukin-1β; IFN-γ: interferon-γ; TGF-β: transforming growth factor-β; ELISA: enzyme-linked immunosorbent assay

## Introduction

*Trueperella pyogenes* (*T. pyogenes*) [1] is an important opportunistic pathogen in livestock, wild animals, and humans [2]. Pyolysin (PLO), which is secreted by all strains of the bacteria [3], contributes significantly to organism pathogenicity [4,5].

PLO is a member of a large group of toxins known as cholesterol-dependent cytolysins (CDCs), which include listeriolysin O, pneumolysin (PLY), suilysin, streptolysin O, intermedilysin, perfringolysin O and arcanolysin [6–12], among others. However, according to previous studies on other CDCs, PLO shares only 31% to 71% similarity with other CDCs in primary structure [3,12]; the low similarity thereby restricts the deduction of the structure, function, and virulence mechanism of PLO. To date, studies to determine the relationship between the structure and function of PLO are limited [5,13–19].

PLY, one of the most extensively studied CDCs, exhibits several biological properties, such as activation of the complement system [20], platelets [21], and NLR family pyrin domain containing protein 3 (NLRP3) inflammasome [22] or stimulation of pattern recognition receptors (PRRs) [23,24]. Studies indicated that the pore-forming activity and pathogen-associated molecular pattern (PAMP) activity of the toxin form the foundation for most of the previously mentioned biological properties. These studies provided valuable information for the current study to investigate the relationship between the function and virulence mechansims of PLO.

The structure of monomeric CDC molecules is divided into four domains (domain 1 to domain 4, D1 to D4) [19]. The pore formation process of CDC molecules can be broken down into three major stages, namely, membrane binding, oligomerization, and formation of the transmembrane β-barrel pore [25]. D4 of CDC molecules contains the initial determinants (conserved undecapeptide and loops L1–L3) and mediates the interaction of the molecules with the membrane; subsequently, D2 and D3 mediate the oligomerization of CDC monomers and the formation of prepore complex; finally, conformational changes in D3 and the structural collapse of D2 lead to the formation of transmembrane pores [25,26]. The role of D2, D3, and D4 in maintaining the hemolytic activity of PLO has been partially investigated [5,13–19]. However, no study has investigated the relationship between D1 and the hemolytic activity of PLO. Furthermore, the linkage between structure and biological functions except for the hemolytic activity of PLO has been poorly studied. A previous study showed that the replacement of certain amino acids in D1 of a molecule with other different amino acids markedly decreased the hemolytic activity of PLY [27], indicating the potential importance of D1 in maintaining the biological function of CDCs. Sequence alignment showed that several amino acids (such as 205th Asp and 339th Asn), which are important for maintaining the hemolytic activity of PLY [27], were highly conserved in CDCs. Thus, PLO mutants constructed from these amino acid sites are useful tools for studying the relationship between D1 and the biological functions of PLO.

By using different PLO mutants, we confirmed in the current study that replacing the 238th aspartic acid (Asp or D) in D1 of PLO with an arginine (Arg or R) impairs the pore-forming activity of PLO and that the pore-forming activity of PLO is essential for inducing excessive inflammation response in mice. The findings provided new information on the relationship between the sturcture and virulence of PLO.

## Results

### rPLO D238R showed impaired hemolytic activity and pore-forming activity in a sRBCs system

**Figure 1.**
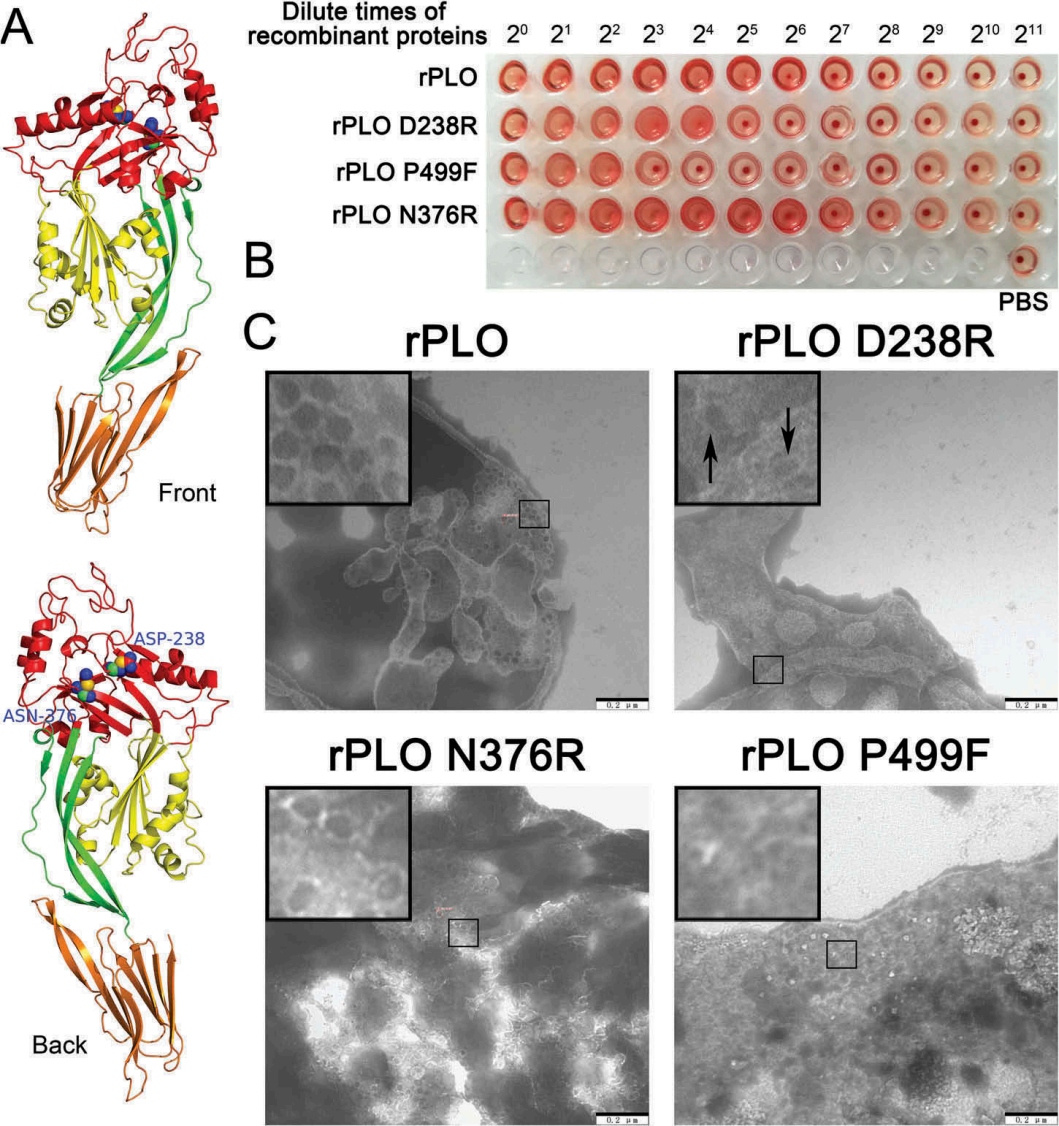
Substitution of the 238th Asp with an Arg impaired the hemolytic and pore-forming activity of PLO. (A) Structure model of PLO molecule and the location of the 238th Asp and the 376th Asn that were replaced with an Arg to construct the PLO mutants. The domains of the PLO molecule were colored red (D1), green (D2), yellow (D3), and orange (D4).(B) Determination of the hemolytic activity of rPLO and mutants. The smallest concentrations of rPLO, rPLO D238R, rPLO P499F, and rPLO N376R that cause complete hemolysis of sRBCs were 3.125, 12.5, 50, and 3.125 μg/mL, respectively. The sRBCs were incubated with PBS as hemolysis-negative controls.(C) Determination of the pore-forming activity of rPLO and the mutants on RBCMs by TEM observation. rPLO and rPLO N376R formed typical pores with diameters of approximately 30 nm. rPLO D238R formed incomplete pores (marked by black arrows). The sRBCs treated with rPLO P499F showed no typical pores on the cell membrane. Scale bar = 200 nm.

Figure 1(A) shows the two substituted amino acids in the current study and the location of the two amino acids in the PLO monomer. As shown in Figure 1(B), rPLO caused complete hemolysis even at an extremely low concentration (3.125 µg/mL). rPLO D238R showed a fourfold decrease in hemolytic activity compared with rPLO, whereas rPLO N376R showed similar hemolytic activity to rPLO. As a negative control, rPLO P499F showed a 16-fold decrease in hemolytic activity compared with rPLO. Quantitative hemolysis assay showed similar results, that is, the hemolytic activities of rPLO D238R, rPLO N376R, and rPLO P499F relative to rPLO were approximately 25%, 100%, and 6%, respectively. TEM observation showed that rPLO and rPLO N376R formed typical pores with 30 nm diameter on the membrane of sRBCs. By contrast, rPLO P499F cannot form pores on sRBCs, whereas rPLO D238R formed pores with oval or irregular shape, indicating that the mutant formed incomplete pores on sheep RBCMs (Figure 1(C)). Given that the basic function of rPLO N376R was similar to that of rPLO, only rPLO D238R was further studied in most of the subsequent experiments.

### rPLO D238R showed similar cell membrane binding capacity to rPLO

**Figure 2.**
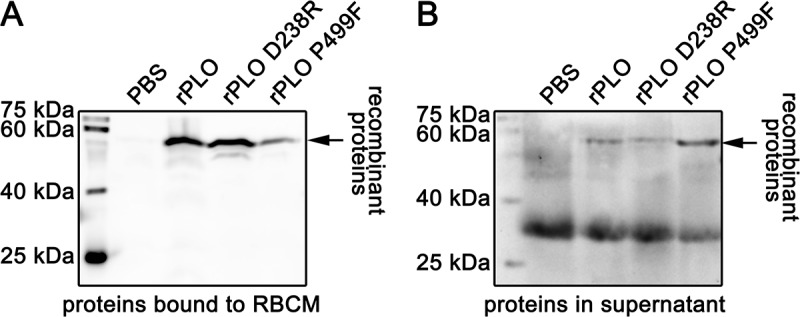
Determination of the cell membrane binding activity of rPLO and mutants by western blot assays. The western blots showed that both rPLO and rPLO D238R can efficiently bind RBCM. rPLO P499F showed impaired cell membrane binding capacity (A). After 30 min of incubation, most of the rPLO P499F proteins were detected in the supernatant (B).

As seen in Figure 2, rPLO D238R can bind to RBCMs with similar efficiency to rPLO. Western blot assay showed that most of rPLO and rPLO D238R bound to RBCMs (Figure 2(A)), and only a small amount of the two proteins was detected in the supernatant (Figure 2(B)). As a negative control, rPLO P499F showed a significant decrease in RBCM binding capacity (Figure 2(A)). After incubation with RBCM for 30 min, most of the rPLO P499F was detected in the supernatant (Figure 2(B)). Bands between 25 and 40 kDa in Figure 2(B) may be attributed to the nonspecific reactive component from RBCMs because the blot showed such a band even when the RBCMs were not been incubated with any of the three recombinant proteins.

Thus, rPLO P499F lost its hemolytic activity owing to failure of binding to the cell membrane, whereas the decrease in hemolytic activity of rPLO D238R can be attributed to the failure of completing the subsequent process after binding to the cell membrane.

### rPLO D238R showed impaired capacity of forming oligomers on cholesterol-containing membrane

**Figure 3.**
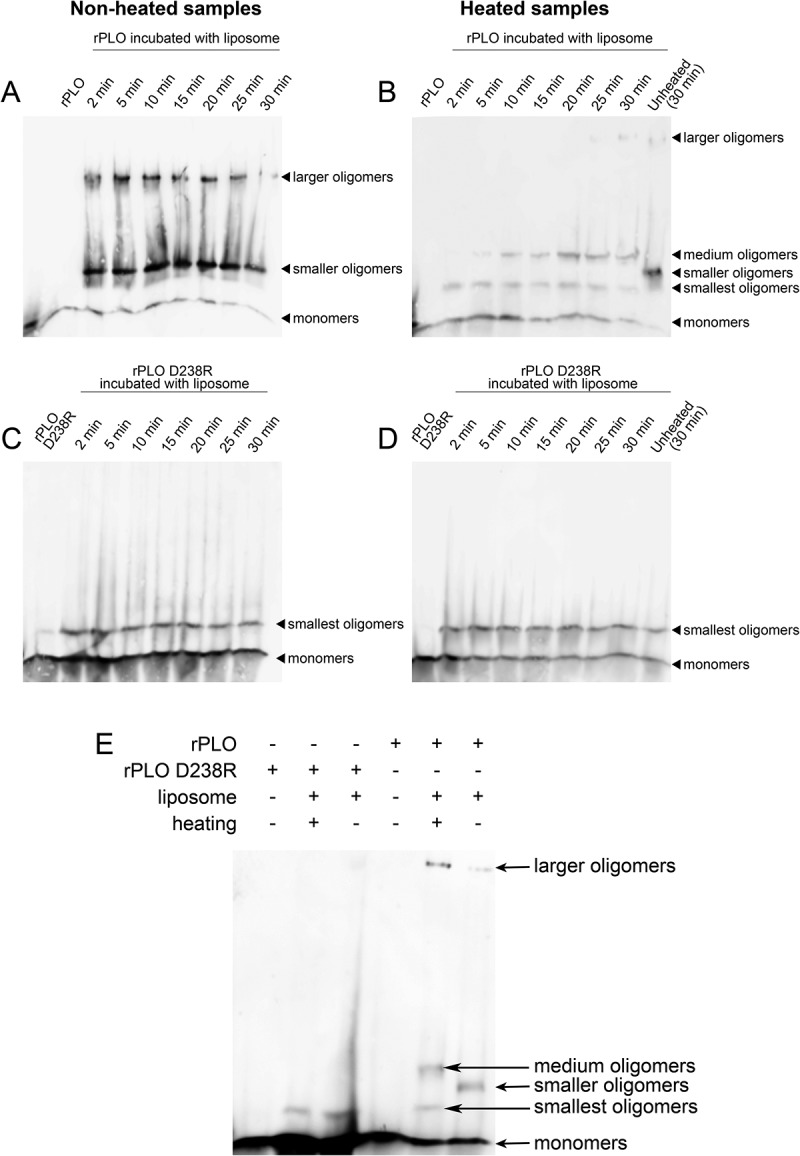
Determination of oligomers formed by rPLO and rPLO D238R by western blot assays. Western blot assays showed that rPLO formed two types of oligomers (large and small) within 2 min when incubated with cholesterol-containing liposomes (A). When samples were heated to 100°C for 10 min before SDS-AGE analysis, the large and small oligomers split into two other types of oligomers (medium and the smallest) (B). rPLO D238R only formed one type of oligomer (C), and heating did not further disrupt the oligomers (D). Analysis of the oligomers formed by rPLO and rPLO D238R in a single Western blot showed that the molecular weight of the only type of oligomer formed by rPLO D238R was similar to that of the smallest oligomers, which can be obtained mainly by heating the large and small oligomers formed by rPLO (E). This result indicated that the smallest oligomers were possibly the intermediate product that further formed the large or small oligomers. The result also indicated that the substitution of the 238th Asp with an Arg mainly hindered the process of large oligomer formation by the smallest oligomers.

As seen in Figure 3(A), western blot assay showed rPLO formed large oligomers after incubation with cholesterol-containing liposomes. Two different kinds of rPLO oligomers were observed (the large and the small oligomers) when after separation using SDS-AGE (Figure 3(A)). rPLO monomers formed oligomers within 2 min after incubation with liposomes (Figure 3(A)). When the samples were heated to 100°C prior to SDS-AGE, the oligomers split into two types of oligomers (medium and the smallest oligomers) (Figure 3(B)).

Different from rPLO, rPLO D238R only formed the smallest oligomers even when the incubating period was extended to 30 min (Figure 3(C)). The smallest oligomers could not be split by heating (Figure 3(D)).

For comparison of the differences between oligomers formed by rPLO and rPLO D238R, the samples were analyzed in a single western blot assay. The molecular weight of the smallest oligomers formed by rPLO was similar to that of the smallest oligomers formed by rPLO D238R (Figure 3(E)), indicating that the smallest oligomer was the basic unit for forming the medium and large oligomers.

### rPLO D238R showed a reduced cytotoxicity in L929 cells and MDBK cells

**Figure 4.**
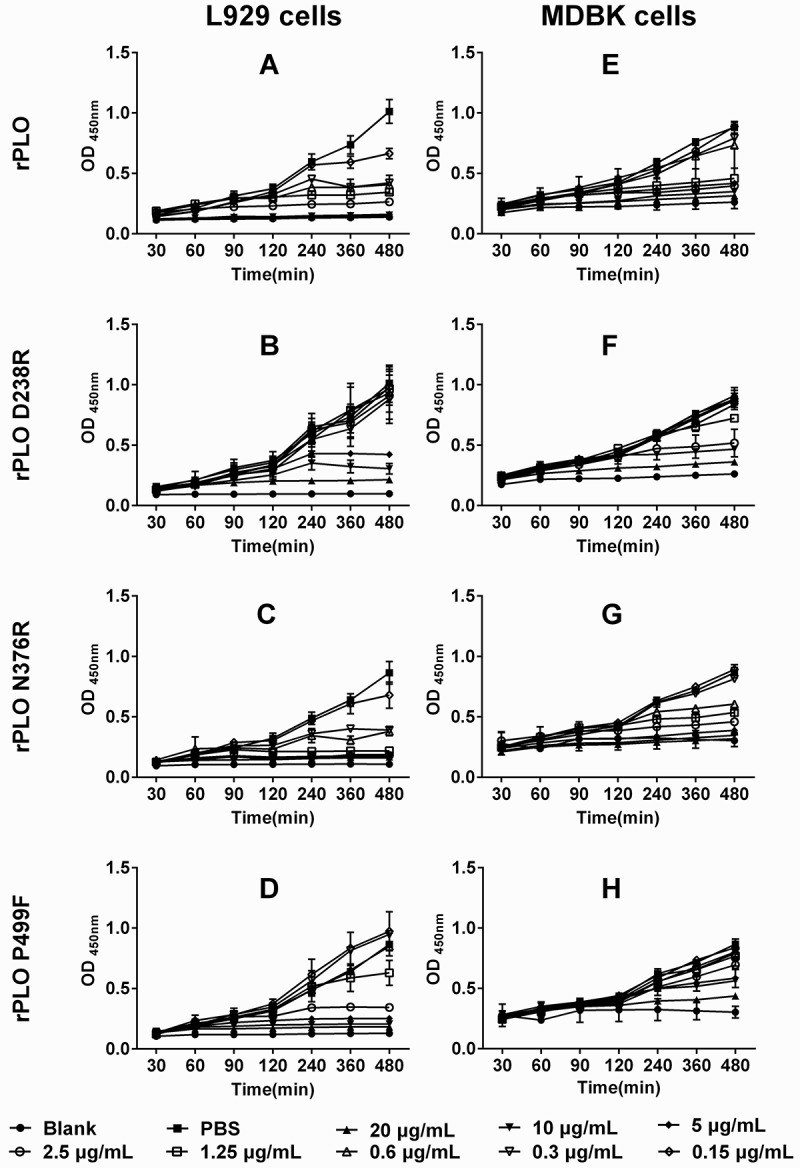
rPLO D238R showed decreased cytotoxicity in L929 and MDBK cells. rPLO and rPLO N376R showed evident cytoxicity in L929 cells even at a low concentration of 0.3 μg/mL (A and C). rPLO D238R did not show evident cytotoxicity in L929 cells at 2.5 μg/mL (B). rPLO P499F was more cytotoxic than rPLO D238R given that 2.5 μg/mL of rPLO P499F evidently decreased the viability of L929 cells (D). Compared with L929 cells, MDBK cells were less sensitive to the cytotoxicity of rPLO and rPLO N376R. rPLO and rPLO N376R showed evident cytotoxic effects in MDBK cells at 1.25 and 0.6125 μg/mL, respectively (E and G). rPLO D238R at 2.5 μg/mL was cytotoxic in MDBK cells (F). rPLO P499F showed cytotoxicity in MDBK cells only when concentration exceeded 5 μg/mL (H).

Even at a low concentration of 0.15 μg/mL, the rPLO and rPLO N376R treatments inhibited the proliferation of L929 cells (Figure 4(A,C)). By contrast, no apparent cytotoxicity was observed in L929 cells treated with 2.5 μg/mL of rPLO D238R (Figure 4(B)). Similar results were observed in MDBK cells (Figure 4(G)). These results indicated that the cytotoxicity of rPLO D238R to L929 cells was significantly lower than that of rPLO.

### Pore-forming activity of PLO was essential for inducing IL-1β expression in L929 cells

**Figure 5.**
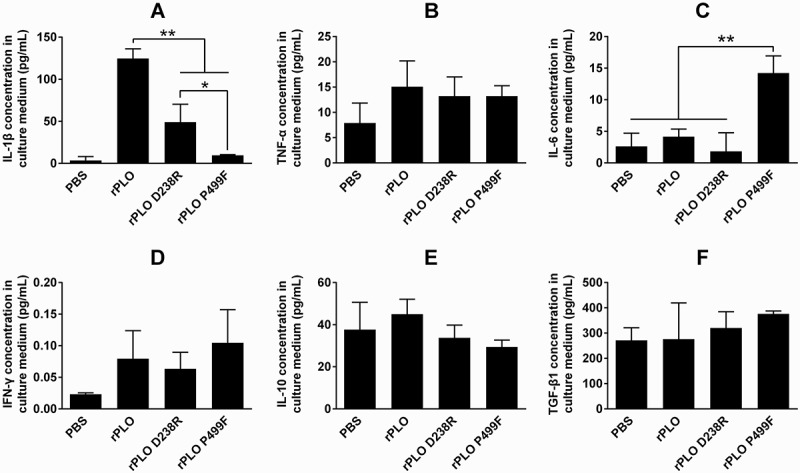
Profile of the expression of inflammation-associated cytokines in L929 cells treated by recombinant proteins. rPLO and rPLO D238R at a concentration of 1.6 μg/mL significantly upregulated the expression of IL-1β within 8 h, but rPLO P499F did not; Meanwhile, the expression level of IL-1β in rPLO-treated cells was significantly higher than that of cells treated with rPLO D238R (A). rPLO P499F upregulated the expression level of IL-6 significantly, whereas rPLO and rPLO D238R showed no such effect (C). The expression levels of TNF-α (B), IFN-γ (D), IL-10 (E), and TGF-β1 (F) did not differ significantly between cells treated with different recombinant proteins (* *p* < 0.05, ** *p* < 0.01).

As shown in Figure 5, at a concentration of 1.6 μg/mL, which is far higher than the sublytic concentration of rPLO (0.1 μg/mL) (Supplemental Figure 1(A)), rPLO and rPLO D238R treatments induced the upregulation of IL-1β expression in L929 cells (Fgiure 5A), and rPLO P499F treatment did not. rPLO induced a significantly higher level of IL-1β than rPLO D238R in L929 cells (Figure 5(A)). rPLO at sublytic concentrations (0.1, 0.05, 0.025, and 0.0125 μg/mL) was used to treat L929 cells did not affect the expression of IL-1β (Supplemental Figure 1(B)). This result indicated that the pore-forming activity and the number of pores formed by rPLO on the cell membrane are crucial for rPLO to induce IL-1β expression in L929 cells. Interestingly, at a concentration of 1.6 μg/mL, rPLO P499F treatment, but not rPLO and rPLO D238R treatments, significantly upregulated the expression of IL-6 in L929 cells (Figure 5(C)). The capacity of rPLO P499F to induce the expression of IL-10 apparently depends on the quantity of the recombinant protein, as observed in rPLO P499F at low concentrations (0.0125–0.1 μg/mL) failing to upregulate the expression of IL-6 in L929 cells (Supplemental Figure 1(C)). The expression levels of TNF-α, IFN-γ, IL-10, and TGF-β1 between cells that received different treatments (1.6 μg/mL of rPLO, rPLO D238R or rPLO P499F) did not differ significantly (Figure 5(B-F)). Concentrations from 0.0125 μg/mL to 0.1 μg/mL of the three recombinant proteins did not affect the expression of IL-10 in L929 cells within 8 h (Supplemental Figure 1(D)).

### rPLO D238R failed to induce excessive inflammation in mice

Mice were intramuscularly inoculated with rPLO, rPLO D238R, or rPLO P499F to investigate the difference in inflammation-inducing capacities between rPLO and the mutants in vivo. At 3 days after the second inoculation, mice inoculated with rPLO and rPLO P499F showed evident skin lesions around the injection sites (data not shown). By contrast, mice inoculated with rPLO D238R or PBS showed no visible skin lesions.

**Figure 6.**
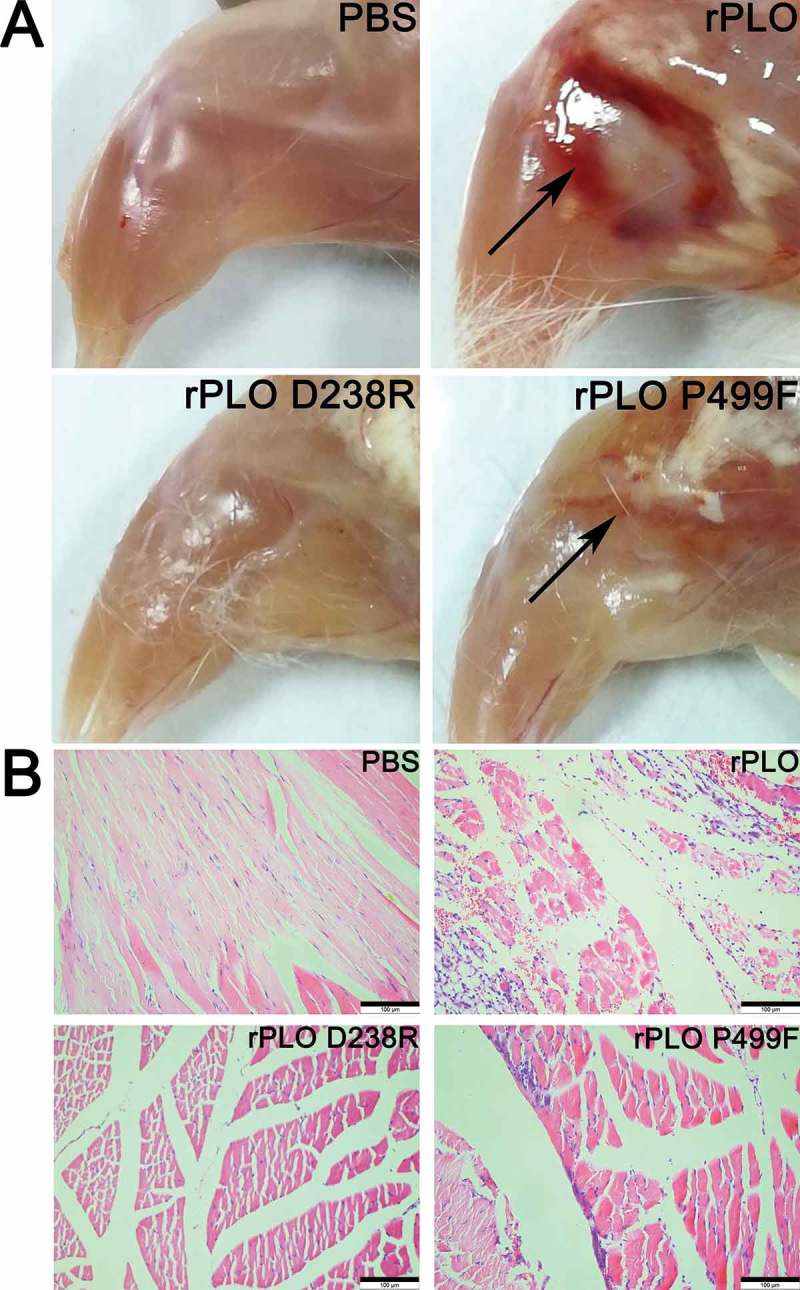
Images and histopathologizal analysis of tissue damage caused by different recombinant proteins in mice. (A) Representative pictures of muscle tissues (hindleg of mice) in the periphery of injection sites of mice inoculated with different recombinant proteins. Mice inoculated with rPLO and rPLO P499F showed visible hemorrhagic damages (marked by black arrows) in the muscle tissues around the injection sites. Mice inoculated with rPLO D238R and PBS showed no visible tissue damage.(B) Histopathological analysis of muscle tissues around injection sites. Massive inflammatory cell infiltration was observed in the tissues of the mice inoculated with rPLO and rPLO P499F but not in the tissues of the other two groups. Scale bar = 100 μm.

The muscles around the injection sites of mice inoculated with rPLO and rPLO P499F showed evident hemorrhagic damage, whereas mice inoculated with rPLO D238R or PBS showed no visible damage in muscles around injection sites (Figure 6(A)). The histopathological examination of the muscle tissues around injection sites showed dense or medium inflammatory cell infiltration in mice inoculated with rPLO and rPLO P499F but not in mice in the other two groups (Figure 6(B)).

**Figure 7.**
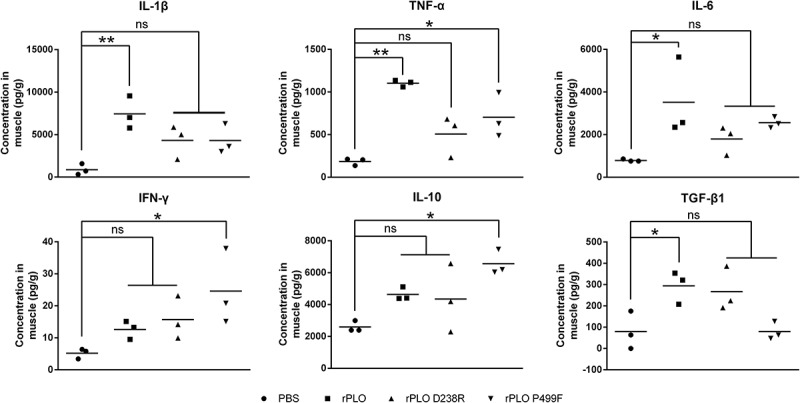
Expression levels of inflammation-associated cytokines in mice inoculated with recombinant proteins. rPLO inoculation significantly upregulated the expression levels of IL-1β, TNF-α, IL-6, and TGF-β1 in mice. rPLO P499F inoculation significantly upregulated the expression of TNF-α, IFN-γ, and IL-10 in mice. None of the six cytokines were significantly upregulated in mice inoculated with rPLO D238R according to statistical analysis. (* *p* < 0.05, ** *p* < 0.01, ns not significant)

ELISA results showed that the expression levels of IL-1β (p < 0.01), TNF-α (p < 0.01), IL-6 (p < 0.05), and TGF-β1 (p < 0.05) in mice inoculated with rPLO were significantly upregulated compared with those of control group mice (Figure 7). Moreover, TNF-α (p < 0.05), IFN-γ (p < 0.05), and IL-10 (p < 0.05) expression levels in mice inoculated with rPLO P499F were significantly higher than those of the control group (Figure 7). The expression levels of the six cytokines in mice inoculated with rPLO P499F were also upregulated. However, the expression levels were not significantly upregulated compared with the levels in the control group (Figure 7). These results indicated that rPLO D238R exhibited an impaired capacity of inducing excessive inflammatory response in vivo.

### rPLO P499F upregulated the IL-10 expression in BMDMs

**Figure 8.**
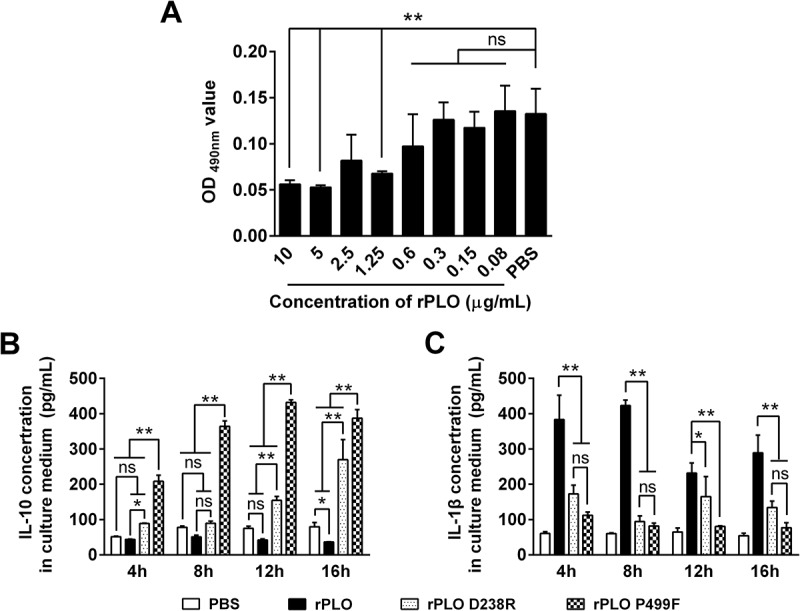
Expression levels of IL-1β and IL-10 in BMDMs treated with rPLO or its mutants at sublytic concentrations. MTT assay showed that rPLO at concentrations lower than 0.6 μg/mL did not signicantly decrease the viability of BMDMs within 16 h (A). Thus, 0.6 μg/mL was defined as the sublytic concentraiton of rPLO in such a system. Furthermore, 0.6 μg/mL rPLO treatment did not significantly affect the expression level of IL-10; by contrast, rPLO P499F treatment significantly upregulated the expression of IL-10 after 4 h, and rPLO D238R elicited signficant upregulation of IL-10 expression after 12 h. However, the expression level of IL-10 elicited by rPLO D238R was significantly lower than that elicited by rPLO P499F at any time point (B). An rPLO concentration of 0.6 μg/mL elicited upregulated IL-1β expression in BMDMs after 4 h, whereas 0.6 μg/mL rPLO P499F did not upregulate the expression of IL-1β even when the incubation period was extended to 16 h. The IL-1β expression elicited by 0.6 μg/mL rPLO D238R was higher than that elicited by rPLO P499F at 12 h but was significantly lower than that elicited by rPLO (C). (* *p* < 0.05, ** *p* < 0.01, ns not significant)

The sublytic concentration of rPLO to BMDMs was initially determined by MTT assay. As shown in Figure 8(A), treatment with rPLO at concentrations lower than 0.6 μg/mL did not significantly decrease the viability of BMDMs within 16 h. Thus, 0.6 μg/mL was used as the working concentration of the rPLO and mutants in the BMDMs system. rPLO P499F induced the upregulation of IL-10 in BMDMs after 4 h of treatment (Figure 8(B)). The concentration of IL-10 in rPLO P499F-treated BMDMs increased to the peak at 12 h and slightly decreased at 16 h, which might be attributed to the decrease in rPLO P499F concentration in the system caused by phagocytosis and the degradation effect of macrophages. However, rPLO P499F treatment did not affect the expression of IL-1β in BMDMs (Figure 8(C)). By contrast, rPLO treatment upregulated the expression of IL-1β in BMDMs but slightly affected the expression of IL-10 (Figure 8(C)). Compared with rPLO and rPLO P499F, rPLO D238R elicited moderate expression levels of IL-10 (Figure 8(B)) and IL-1β (Figure 8(C)), respectively.

## Discussion

Although numerous studies have determined that CDCs can interact with host cells in different manners, such recognition by PRRs [23,24] or B-cell receptors, the intrinsic cell membrane binding capacity may drive these toxins to preferentially bind to the cell membrane by themselves. Thus, the effects caused by CDCs are likely attributed to their intrinsic pore-forming function but not to their properties as ligands. Therefore, investigating the relationship between the pore-forming function of CDCs and the virulence mechanism of these toxins is of practical value.

Molecular structure is essential in exhibiting the function of a given protein. As other CDCs, the structure of PLO molecules can be divided into four domains [19]. Three of the four domains possess a unique capacity to aid CDC molecules to form pores. D4 in CDCs serves as an anchor to cholesterol-containing membranes, D3 forms β-barrel structures of the pore after undergoing massive rearrangement and refolding, and D2 connects D1 to D4 and collapses to allow D3 to insert into cholesterol-containing membranes [28]. However, D1 in CDCs is not extensively characterized. One of our previous studies showed that antibodies targeted to epitopes located in D1 of PLO exhibit antihemolytic activities [16]. On the basis of this finding, we speculated that D1 played an essential role in forming pores, at least for PLO.

A previous study [27] showed that replacement of the 205th Asp or 339th Asn with an Arg significantly impairs the hemolytic activity of PLY. Hence, the 205th Asp and 339th Asn of PLY are important for maintaining the primary biological function or properties of PLY. Sequence alignment showed that the Asp and Asn are conserved in PLO. In PLO, the homologous Asp and Asn are located at the 238th and 376th positions, respectively. The 238th Asp and 376th Asn are spatially close to our previously observed epitope [16].

In the current study, we first constructed two PLO mutants, each with a single amino acid substitution in D1 (Figure 1(A)). Based on the PLO molecular model (Figure 1(A)), the two substituted amino acids were spatially near to each other; however, the 238th Asp was located in the nonregular coil linking D1 and D3 of PLO, whereas the 376th Asn was located in the nonregular coil that linked the D1 and D2 of PLO. The 238th Asp and the 376th Asn evidently played different roles in the PLO pore formation. rPLO D238R partially lost its hemolytic activity, whereas rPLO N376R showed no impairment in hemolytic activity compared with rPLO. The results in Figures 1(C) and 3 show that rPLO D238R formed the smallest oligomers and incomplete pores in the membrane. Substitution of the 238th Asp with an Arg possibly rendered the large oligomers unstable and consequently decreased their efficiency to form complete pores. Thus, the substitution of 238th Asp with an Arg mainly disturbed the assembly of large PLO oligomers by the smallest PLO oligomers.

*T. pyogenes* infection generally leads to different types of inflammatory diseases, such as arthritis, endocarditis, mastitis, pneumonia, and osteomyelitis in animals [3]. However, the relationship between PLO and the inflammatory diseases caused by *T. pyogenes* infection has not been clearly defined. Jost et al. reported that PLO upregulates the TNF-α expression in J774A.1 macrophages [3]. In the current study, rPLO treatment upregulated the expression of IL-1β in fibroblast cells (L929 cells) (Figure 5(A)). This upregulation indicated that PRR-independent mechanisms were engaged by PLO to elicit the upregulation of IL-1β expression. IL-1β expression in rPLO-treated L929 cells possibly depended on the pore-forming activity of PLO given that rPLO mutants (rPLO P499F and rPLO D238R) with impaired pore-forming activity induced a significantly low level of IL-1β (Figure 5(A)). Another evidence that supports this speculation is that rPLO at a sublytic concentration (lower than 0.1 μg/mL) failed to elicit the expression of IL-1β in L929 cells (Supplemental Figure 1(A)). These data also indicated that a sufficient number of pores formed by rPLO was important for eliciting the expression of IL-1β in L929 cells. Amos et al. found that 0.003 to 30 hemolytic units of PLO did not stimulate the expression levels of IL-1β, IL-6, and IL-8 in bovine endometrial or hematopoietic cells or in vitro organ cultures of the endometrium [13]. The difference between the results of Amos’s and our studies can be attributed to the different types of cells employed. In the current study, rPLO and rPLO D238 treatments failed to upregulate the expression level of IL-6 in L929 cells. By contrast, rPLO P499F significantly upregulated the expression of IL-6 in the cultured cells (Figure 5(C)). This finding indicated that the other biological activities of PLO molecules may emerge only when the molecules lose their cell membrane binding capacity or the molecules lack opportunities to reach the cell membrane. This behavior can cause the higher toxicity of rPLO P499F compared with rPLO D238R in L929 cells (Figure 4).

The animal experiments demonstrated that rPLO possessed the largest potential to induce the expression of proinflammatory cytokines in vivo (Figure 7). By contrast, rPLO D238R failed to significantly upregulate the expression of any of the six cytokines in vivo (Figure 7). These data indicate that PLO pore-forming activity is essential to induce inflammatory responses in vivo. PLY, another CDC, induces the upregulation of proinflammatory cytokines through numerous mechanisms, such as inducing cell necroptosis [29] and pyroptosis [30], promoting platelet activation and platelet–neutrophil interactions [31], initiating the transcription of genes of proinflammatory cytokines [32], and activating inflammasomes to promote the maturation of certain proinflammatory cytokines by changing the intracellular ion concentration [22]. Most of these events are related to the pore-forming activity of PLY. In the current study, rPLO P499F also tended to elicit the expression of certain inflammation-associated cytokines in mice, but the cytokine expression profile was markedly different from that in rPLO-treated mice (Figure 7). These data indicates that rPLO P499F functions in a significantly different manner from rPLO in mice. Interestingly, rPLO P499F treatment, not rPLO and rPLO D238R treatments, significantly upregulated IL-10 expression in vivo compared with PBS treatment (*p* < 0.05). PLY is one of the the ligands of Toll-like receptor 4 (TLR4) [23,24], and TLR4 stimulation can upregulate the expression of IL-10 in macrophages [33]. Thus, this study speculated that rPLO P499F possibly induces an increased IL-10 expression, at least partially, in this manner. BMDMs were used to comfirm this speculation. rPLO treatment can upregulate the expression of IL-1β (Figure 8(C)), but not IL-10 (Figure 8(B)), in BMDMs; rPLO P499F significantly upregulated the expression of IL-10 (Figure 8(B)) but showed a slight effect on the expression of IL-1β (Figure 8(C)). By contrast, rPLO D238R induced moderate expression of IL-10 (compared with rPLO P499F) (Figure 8(B)) and IL-1β (compared with rPLO) (Figure 8(C)) in BMDMs. The results obtained from BMDMs agreed with the results of animal experiments (Figure 7) in this study. Thus, this study speculated that the difference in cytokine expression profiles between mice inoculated with rPLO and rPLO P499F is attributed to larger opportunity for rPLO P499F molecules in comparison with rPLO and rPLO D238R molecules to be recognized by the PRRs of immune cells, such as macrophages and neutrophils; the difference in opportunity is due to the impaired cell membrane binding capacity of rPLO P499F. On the one hand, the results confirmed that PLO induced the expression of IL-1β mainly through a pore-forming-dependent mechanism, as observed in L929 cells. On the other hand, the results also indicated that rPLO P499F induced the cytokine expression through a PAMP-activity-dependent mechanism. However, PLO molecules in infected animals encounter the cholesterol-containing host cell membrane immediately after being secreted by *T. pyogenes*. Therefore, from our perspective, the effects elicited by the other biological activities of PLO did not outweigh the effects elicited by the pore-forming activity of PLO molecules in vivo.

In conclusion, the current study demonstrated that replacing the 238th Asp, which is located in D1 of PLO, with an Arg impaired the pore-forming activity of PLO. Then, by using the mutants of PLO with impaired pore-forming activity, the pore-forming activity and not the PAMP property of PLO was confirmed to be responsible for eliciting an excessive inflammation response.

## Materials and methods

### Construction and expression of recombinant PLO (rPLO) and rPLO mutant

A recombinant plasmid containing the *plo* gene without the nucleotide-encoding signal peptide was prepared in the laboratory and named pET-30a-plo [34]. Recombinant plasmids pET-30a (+)-plo P499F, pET-30a (+)-plo D238R, and pET-30a (+)-plo N376R were constructed using a PCR-mediated DNA mutation system, denoted by Fast Mutagenesis System (TransGen Biotech, Beijing). The recombinant plasmid pET-30a-plo was used as template, and the sequence of the primers are listed in Table 1. The resulting pET-30a (+)-plo P499F encoded rPLO P499F with proline (P) at position 499 of immature PLO replaced with phenylalanine (F). rPLO P499F (named His-PLO.F_499_ in Billington [35]) reportedly lost its cell membrane binding capacity compared with rPLO and was used as a control in the current study. pET-30a (+)-plo D238R and pET-30a (+)-plo N376R encoded rPLO D238R and rPLO N376R, respectively. For rPLO D238R, the Asp at position 238 of rPLO was replaced with arginine (Arg or R), whereas for rPLO N376R, asparagine (Asn or N) at position 376 of rPLO was replaced with Arg.

**Table 1.** Sequence of the PCR primers for constructing the mutants of *plo* gene.

Name of primer	Sequence	Mutation site
*plo-*P499F(F)	5ʹ-GGCCTAGCGTGGGATTTCTGGTGGACCGTT-3’	Pro (499) to Phe
*plo*-P499F(B)	5ʹ-GATAACGGTCCACCAGAAATCCCACGCTA-3’	
*plo*-D238R(F)	5ʹ-TACACGGCTAGCGTACGTACACCGACATCT-3’	Asp (238) to Arg
*plo*-D238R(B)	5ʹ-ACGTACGCTAGCCGTGTAGTAAATCTGTTT-3’	
*plo*-N376R(F)	5ʹ-AATTTCTTGAAGGATCGTCAGTTGGCAGCT-3’	Asn (376) to Arg
*plo*-N376R(B)	5ʹ-ACGATCCTTCAAGAAATTGACAGCATAGGA-3’	

All recombinant plasmids were transformed into *Escherichia coli* Rosetta (DE3)^TM^ competent cells. Isopropyl-β-d-thiogalactoside (IPTG) was used to induce the expression of recombinant proteins rPLO, rPLO P499F, rPLO D238R, and rPLO N376R. Then, the proteins were purified using nickel-charged resin and dialyzed against phosphate-buffered saline (PBS) with 5% glycerol at 4°C for 48 h. The proteins were subsequently quantified using Bradford method and stored at −80°C until use.

### Hemolysis assay

Assays for detecting the hemolysis capability of rPLO and its mutants were performed as described previously [17]. rPLO and mutants were first adjusted to a concentration of 100 μg/mL. Then, the proteins were serially diluted twofold with PBS. A total of 50 μL of diluted proteins was added into each well of V-bottomed 96-well microtiter plates. Then, 50 μL of a 2% suspension of sheep red blood cells (sRBCs) was added to each well of the microtiter plates. The mixtures of proteins and sRBCs were incubated at 37°C for 30 min.

For quantitatively analyzing hemolytic assay results, 100% and 0% hemolysis controls were initially prepared. In brief, 1 mL of sRBCs (1%) in PBS was either ultrasonically disrupted (200 W, 5 s for five times at intervals of 5 s) on ice or incubated at 37°C for 30 min. Subsequently, the mixtures were centrifuged (1500*g*) at 4°C for 10 min. The absorbance of the supernatant from the ultrasonically treated sRBCs at 450 nm was defined as the value representing 100% hemolysis in such a system, whereas the absorbance at 450 nm of the supernatant from 37°C-treated sRBCs was defined as the value representing 0% hemolysis. Next, 100 μL of serially twofold-diluted rPLO or its mutants was mixed with 100 μL of sRBCs (2%) (with a final sRBC concentration of 1%). The mixtures were incubated at 37°C for 30 min and then centrifuged (1500*g*) at 4°C for 10 min. The supernatant (100 μL) of each sample was transferred into a flat-bottomed 96-well microtiter plate, and the absorbance of each well was measured at 450 nm. The amount of recombinant proteins that caused 50% hemolysis was defined as 1 hemolytic unit. The relative hemolytic activity of the mutants was expressed as a percentage of the rPLO hemolytic activity, which was set at 100%.

### Transmission electron microscopy (TEM) observation

rPLO and mutants were adjusted to a concentration of 400 μg/mL. Then, 100 μL rPLO and the mutants were added separately into 900 μL 2% sRBC. The mixtures were incubated at 37°C for 30 min and subsequently centrifuged at 5000 r/min for 7 min at 4°C, and the supernatant was discarded. Then, the obtained precipitates were resuspended in 200 μL fresh PBS, and specimens were transferred to electron microscopy grids and stained with 1% (wt./vol.) uranyl acetate. The negatively stained specimens were examined using a Hitachi H-7650 electron microscope at an acceleration voltage of 100 kV.

### Membrane binding assay

Assays for detecting the membrane binding and hemolytic capabilities of rPLO and the mutants were performed as described previously [17]. In brief, sheep red blood cell membrane (RBCM) was prepared by suspending prepared sRBCs in deionized water for 4 h to 6 h at 4°C and then centrifuging the mixture at 5000 r/min for 10 min at 4°C. The cell membrane precipitate was resuspended in PBS. rPLO or the mutants were incubated with the cell membrane suspension for 30 min at 37°C. Then, the mixtures were separated into supernatant and precipitate by centrifugation at 12000 r/min for 10 min at 4°C. Precipitates were washed twice with PBS. The components in the supernatant were precipitated by trichloroacetic acid addition. The precipitates were redissolved in NaOH. All samples were separated using 12% sodium dodecyl sulfate–polyacrylamide gel electrophoresis (SDS-PAGE) and transferred onto a nitrocellulose (NC) membrane. Then, Western blot was conducted using mouse anti-Histidine (His) tag monoclonal antibodies.

### Determination of the oligomerization of rPLO and the mutants by sodium dodecyl sulfate agarose gel electrophoresis (SDS-AGE)

Liposome was prepared. In brief, 0.281 g of lecithin and 0.09 of g cholesterol were dissolved in 20 mL of ether and injected gradually by using a 5 mL syringe into 20 mL of PBS heated to 30°C. The mixture was stirred for 10 min at room temperature and then heated to 55°C for 1.5 h. The suspension was ultrasonicated for 10 min (5-s treatment with intervals of 3 s). Subsequently, the suspension was passed through a 0.45 μm filter. The product was a liposome suspension and stored at 4°C.

A total of 120 μL of rPLO or mutants was mixed with 120 μL of liposomes and then incubated at 37°C for 30 min. At 2, 5, 10, 15, 20, 25, and 30 min, partial mixtures were harvested. The mixtures were added with SDS-PAGE loading buffer without dithiothreitol. The samples were heated to 100°C for 10 min immediately in certain experiments; in other experiments, the samples were not heated and stored at 4°C until all samples were harvested.

SDS-AGE was conducted using 1.0% (w/v) agarose gel. The 0.2 g agarose was first dissolved in 20 mL of TAE buffer. Then, 200 μL of 10% SDS was added into the solution to prepare the agarose gel. The previously harvested samples were separated using SDS-AGE (180 V, 60 min) and transferred to an NC membrane (75 mA, 120 min). Then, Western blot was conducted using mouse anti-His tag monoclonal antibodies.

### Cytotoxicity assay of rPLO and the mutants in cultured cells

L929 cells or Madin–Darby bovine kidney (MDBK) cells were cultured in a complete culture medium, which contained Roswell Park Memorial Institute-1640 (RPMI-1640) medium with 10% fetal bovine serum (FBS). Cells were seeded in a 96-well cell culture plate at a density of 1 × 10^4^ cells/well and allowed to attach for 8 h. Then, the culture medium was discarded, and 98 μL of fresh complete culture medium and 2 μL of rPLO or the mutants were added into each well. The final concentration of rPLO or mutants ranged from 0.15 μg/mL to 20 μg/mL. Control cells were treated with 98 μL of fresh complete culture medium plus 2 μL of PBS and cultured at 37°C. Cell viability was determined using Cell Counting Kit-8 (C0037, Beyotime)

### Inflammation-associated cytokine induction assay in L929 cells and mouse bone marrow-derived macrophages (BMDMs)

Sublytic concentrations of rPLO to L929 cells and BMDMs were determined by CCK8 or MTT assays. Sublytic concentrations of rPLO to L929 cells and BMDMs were defined as the highest concentrations of rPLO that cannot significantly decrease the viabilities of L929 cells and BMDMs within 8 and 16 h, respectively.

L929 cells were cultured in complete culture medium. Cells were seeded in 6-well cell culture plates at a density of 1 × 10^6^ cells/well and allowed to attach for 8 h. Then, the culture medium was discarded, and 990 μL of fresh complete culture medium and 10 μL of rPLO or the mutants were added into each well. Control cells were treated with 990 μL of fresh complete culture medium plus 10 μL PBS and then cultured at 37°C. At 8 h post-treatment, the supernatant was harvested and stored at −80°C prior to use in detecting inflammation-associated cytokines.

BMDMs were obtained from C57BL/6 mice (3–6 weeks old). Bone marrow was flushed from mice femurs after sacrifice. The cells were washed and resuspended in RPMI-1640 medium with 10% endotoxin-free FBS and 15% (v/v) L929-cell-conditioned medium, which served as a biological source of M-CSF. The medium was replaced twice on days 3 and 5, and nonadherent cells were removed. The cells were used for experiments after day 7, reseeded in a 24-well cell culture plate at a density of 2.5 × 10^5^ cells/well, and allowed to attach for 8 h. Subsequently, the culture medium was discarded, and 500 μL of fresh complete culture medium containing a sublytic concentration of rPLO or each mutant at an equal concentration to rPLO was added. The cells treated with fresh complete culture medium plus PBS served as control. Then, cells were cultured at 37°C, and the supernatant was harvested and stored at −80°C at 4, 8, 12, and 16 h after treatment prior to use in detecting inflammation-associated cytokines.

### Animal experiments

The experimental protocol on animals was approved by the Ethics Committee on the Use and Care of Animals, Northeast Agricultural University on the basis of the Guide for the Care and Use of Laboratory Animals (Institute of Laboratory Animal Resources, Commission on Life Sciences, National Research Council, 2000).

A total of 12 six-week-old female BALB/c mice were randomly divided into four groups. On days 0 and 7, mice in the three experimental groups received intramuscular injections of 50 μg of rPLO, rPLO D238R, and rPLO P499F, respectively, in a final volume of 300 μL. Mice in the fourth group received intramuscular injections of PBS containing 5% glycerol. The animal subjects were humanely sacrificed on day 4 after the second inoculation. Muscle tissues near injection sites were collected for histopathological study and inflammation-associated cytokine detection. For inflammation-associated cytokine detection, tissues were weighed and homogenized in sterile PBS on ice. Then, homogenates were ultrasonically treated for 10 min. The samples were centrifuged at 12000 r/min at 4°C for 5 min. Then, the supernatants were harvested and stored at −80°C for a maximum of 1 month until use in detecting inflammation-associated cytokines.

### Detection of inflammation-associated cytokines

The expression levels of six inflammation-associated cytokines, tumor necrosis factor α (TNF-α), interleukin-1β (IL-1β), IL-6, interferon-γ (IFN-γ), IL-10 and transforming growth factor-β (TGF-β), in cell culture medium and homogenate supernatants were detected by using enzyme-linked immunosorbent assay (ELISA) kits (Neobioscience Technology Co., Ltd., China) according to the manufacturers’ instructions. The amount of cytokines in homogenate supernatants were converted to the amount of cytokines in 1 gram of muscle tissue for further statistical analysis.

### Histopathological study

For histopathological study, collected tissues were fixed in 10% formaldehyde solution. The specimens were paraffin embedded, sectioned, and stained with hematoxyline-eosin. The tissues were visualized with a Leica DM2000 microscope under 10× magnification. Images were then captured using Leica IMS500 software.

### Statistical analysis

One-way ANOVA test was enrolled (Prism, GraphPad Software). The data were presented as mean and SD. A p value < 0.05 was considered significant (* *p* < 0.05, ** *p* < 0.01).
