# Supplementary material for: Replacing the 238th aspartic acid with an arginine impaired the oligomerization activity and inflammation-inducing property of pyolysin
Source: Virulence. 2018 Aug 1;9(1):1112–25. doi: 10.1080/21505594.2018.1491256 (PMC6086297; doi:10.1080/21505594.2018.1491256)
Supplement: Supplemental Material [file kvir-09-01-1491256-s001.zip › Supplemental Figure 1-caption.docx]

Supplemental Figure 1. rPLO and mutants at sublytic concentrations failed to elicit the expression of IL-1β, IL-6, and IL-10 in L929 cells.

rPLO at concentrations lower than 0.1 μg/mL did not cause significant decrease in viability of L929 cells within 8 h (A). Thus, the sublytic concentration of rPLO in this system was defined as 0.1 μg/mL. rPLO, rPLO D238R, and rPLO P499F at concentrations of 0.1, 0.05, 0.025, and 0.0125 μg/mL failed to stimulate the expression of IL-1β (B), IL-6 (C), and IL-10 (D) in L929 cells.
